# Triphala: current applications and new perspectives on the treatment of functional gastrointestinal disorders

**DOI:** 10.1186/s13020-018-0197-6

**Published:** 2018-07-18

**Authors:** Aleksandra Tarasiuk, Paula Mosińska, Jakub Fichna

**Affiliations:** Department of Biochemistry, Faculty of Medicine, Medical University of Lodz, Mazowiecka 6/8, 92-215 Lodz, Poland

**Keywords:** Triphala, Ayurvedic medicine, Irritable bowel syndrome

## Abstract

**Background:**

Ayurvedic medicine is based on natural healing methods that use herbal medicine to cleanse the body of toxins and to attain physical and mental regeneration. Triphala (TLP) is one of the most important ayurvedic supplements and is believed to have a beneficial effect on the entire gastrointestinal (GI) tract.

**Purpose:**

We aim to summarize available literature focused on the components of TLP (*Terminalia chebula*, *Terminalia bellerica* and *Phyllanthus emblica*) and discusse their effectiveness and therapeutic value for improving lower GI symptoms in functional GI disorders, particularly irritable bowel syndrome (IBS).

**Methods:**

This study is based on pertinent papers that were retrieved by a selective search using relevant keywords in PubMed and ScienceDirect databases.

**Results:**

The components of TLP are believed to cause restoration of the epithelium lining of the digestive tract, and by exhibiting mild laxative properties facilitate passage of stool in the colon. TLP is rich in polyphenols, vitamin C and flavonoids, which provide antioxidant and anti-inflammatory effects. It also contains various types of acids, such as gallic, chebulagic and chebulinic, which additionally possess cytoprotective and antifungal properties.

**Conclusion:**

Triphala holds potential in improving lower GI symptoms and may be a valuable and effective addition to standard treatment of IBS. Supplementation of TLP herbal formulations alone or along with other probiotics can be recommended in ongoing clinical studies.

## Background

The pathogenesis of primary intestinal motility disorders is believed to be multifactorial, but neither biochemical nor structural abnormality has been displayed regularly, other than in some forms of intestinal pseudo-obstruction. Patients with mild-to-moderate gastrointestinal (GI) motility disturbances often exhibit peripherally generated symptoms with gut-based characteristics, e.g. intermittent, crampy abdominal pain, indigestion, nausea, bloating or feeling of incomplete evacuation, whereas others experience noxious, persistent and severe symptoms with marked GI tract function impairment, usually accompanied by somatic-psychiatric comorbidities or extra-intestinal symptoms, such as headache, musculoskeletal pain, dizziness or fatigue.

Irritable bowel syndrome (IBS) is the most common type of functional GI disorders (FGID) [1] and is characterized by recurrent abdominal pain and disordered bowel habits. IBS patients represent almost 50% of all individuals who seek medical assistance with primary care physicians or GI specialists [2]. There is no specified marker for IBS and its diagnosis is symptom-based.

The cause of IBS development remains undefined, which makes it difficult to diagnose and treat. Recent studies highlight the interaction between luminal variables (e.g. foods and microbes dwelling in the digestive system), the epithelial barrier, and the mucosal immune system, as a complex network involved in the pathogenesis of IBS [3, 4]. The overstimulation of the mucosal immune system prompts the infiltration of mucosal immunocytes, especially mast cells (MCs), and promotes secretion of various mediators, such as proteases, histamine, and prostanoids, which further propagate permeability dysfunction and activate atypical neural reactions involved in abdominal pain perception and gut motility [3].

With respect to a wide variety of symptoms seen in IBS, patients tend to use different methods for symptom relief, including alternative treatments, such as acupuncture or dietary supplements. Usually, changing nutritional regimen and lifestyle are the first steps taken by patients mainly because such changes can be incorporated into daily life from 1 day to another, without an in-depth clinician’s consultation. An extensive research has provided convincing evidence for beneficial effect of nutritional supplements, including the intake of probiotics, prebiotics or symbiotics, and herbal medicine products on the gut microenvironment. Herbal medicines have been widely used for the treatment of IBS patients [5]. Probiotics and dietary supplements can normalize bowel movements, reduce visceral hypersensitivity and by stimulating the immune system reduce inflammation and gut permeability [6–8]. Alternative treatments still warrant scientific validation of their efficacy.

## Phytochemical constituents of Triphala (TLP)

Triphala is an ayurvedic herbal formulation of dried fruits from three herbal plants in equal proportions: *Terminalia chebula* (black myrobalan), *Terminalia bellerica* (bastard myrobalan) and *Phyllantus emblica* (emblic myrobalan or Indian gooseberry) [9, 10] (Table 1). Fruits of *T. chebula*, that are harvested in the spring, are a rich source of tannins (30–40%), e.g. chebulic acid, chebulinic acid, neochebulinic acid, corilagin, chebulagic acid, gallic acid, ellagic acid, punicalagin, terchebin and terflavin A (Table 2). *T. chebula* fruits additionally contain flavonoids (luteolin, rutins and quercetin), and other phytochemicals (anthraquinones, saponins, β-d-glucogallin, 1, 3, 6-trigalloyl glucose, and 1, 2, 3, 4, 6-penta-*O*-galloyl), starches, amino acids (aspartic acid, glutamic acid, arginine, proline and lysine), fructose, succinic acid, β-sitosterol [39] and fatty acids [40, 41]. So far, *T. chebula* fruits have been utilized in conventional pharmaceuticals to battle ailments of the upper respiratory tract, GI tract, urinary tract and skin [42].

**Table 1:** The main constituents of Triphala and their potential therapeutic effects

Botanical (Hindi name, English)	Phytochemicals	Percentage of phytochemical in each Triphala constituent^a^	Indication for therapeutic usage	References
*Terminalia chebula* (Harad, Chebulic Myrobalan)	Gallic acid	0.024% (w/w)	Constipation, hemorrhoid, skin disease, asthma, dysentery, uterine debility, anemia, diabetes, leukoderma, tumors and heart disease	[1–3]
	Tannic acid	0.011% (w/w)		
	Syringic acid	0.009% (w/w)		
	Epicatechin	0.006% (w/w)		
	Ascorbic acid	0.020% (w/w)		
	Chebulinic acid	Unknown		
	Anthraquinone	Unknown		
	Phosphoric acid	Unknown		
*Terminalia bellerica* (Baheda, Belliric Myrobalan)	Gallic acid	0.005% (w/w)	Cough, asthma, anorexia, vomiting, arthritis, fever, epilepsy, splenomegaly, piles, diarrhea, leprosy, brain tonic and laxative	[2–6]
	Tannic acid	0.004% (w/w)		
	Ascorbic acid	0.023% (w/w)		
	β-sitosterol	Unknown		
	Ellagic acid	Unknown		
	Chebulic acid	Unknown		
	Mannitol	Unknown		
	Oxalic acid	Unknown		
	Galloyl	Unknown		
	Galactose	Unknown		
	Fructose	Unknown		
*Phyllanthus emblica* (Amala, Emblic Myrobalan)	Ascorbic acid	0.036% (w/w)	Diabetes, hysteria, jaundice, eczema, piles, diarrhea, menorrhagia, scurvy, rebuilds and maintains new tissues and increases red blood counts	[3, 7–11]
	Gallic acid	0.081% (w/w)		
	Nicotinic acid	Unknown		
	Ellagic acid	Unknown		
	Linoleic acid	Unknown		
	Linolenic acid	Unknown		
	Oleic acid	Unknown		

^a^Dried fruits of *T. chebula*, *T. bellirica*, and *P. emblica* were purchased from Charoensuk traditional manufacturing, Nakorn Pathom province and ground to powder, then, prepared in the ratio 1:1:1 [3]

**Table 2:** Major phytochemicals of Triphala constituents

Phytochemical	Structural formula	Properties	Refs.
Gallic acid		Inhibits neuronal death; Exerts anti-cancer properties against leukemia, colon and certain prostate cancers, and lung cancer cells; Prevents cellular mutations; Does not affect healthy cells	[1, 2, 12–14]
Chebulic acid		Free radical scavenging activity in vitro; ferric-reducing antioxidant activity; significantly reduces cell cytotoxicity Promising intervention agent against diabetic vascular complication;	[2, 4–6, 15–18]
Chebulinic acid		Anti-inflammatory activity; Natural inhibitor of vascular endothelial growth factor-a mediated angiogenesis	[17, 19]
Ellagic acid		Neuroprotective effect;	[17, 20, 21]
Tannic acid		Astringent property due to the presence of polyphenolic groups; Used as a treatment for many toxic substances, such as strychnine, mushroom, and ptomaine poisonings in the late 19th and early 20th centuries	[11, 12, 22–25]
Epicatechin		Acts as an antioxidant in high concentration in vitro; Prevents cisplatin-induced apoptosis, intracellular reactive oxygen species generation and mitochondrial dysfunction	[26–29]
Syringic acid		Antibacterial and antioxidant effect	[11, 12, 23–25, 30]
Ascorbic acid		Reducing agent and scavenger of radicals (sink of radicals); excellent source of electrons, donates electrons to free radicals such as hydroxyl and superoxide and quenches their reactivity	[30–38]

*Terminalia bellerica* fruits contain mainly proteins (40%) and oils (35%), including omega-3 and -6 fatty acids (e.g. linoleic acid). Because of the high proportion of fatty acids, this plant may influence cholesterol level i.e. increase the level of HDL and decrease LDL, and simultaneously be useful in the treatment of coronary artery disease [43].

Although the specific contents of *P. emblica* (commonly known as amla) are disputed, the fruits are high in ascorbic acid (vitamin C), reaching up to 445 mg per 100 g [38]. The overall bitterness of amla possibly originates from the high density of ellagitannins (diverse class of hydrolysable tannins), such as emblicanin A (37%), emblicanin B (33%), punigluconin (12%) and pedunculagin (14%) [33]. These fruits are also a source of punicafolin and phyllanemblinin A, phyllemblin and other polyphenols, such as flavonoids, kaempferol, ellagic acid, and gallic acid [10, 38].

### TLP as source of tannins, phytosterols, and flavonoids

HPLC analysis revealed that the most commonly occurring polyphenolic compounds in TLP are: phenolic acids (gallic acid, tannic acid, syringic acid and epicatechin), flavonoids and tannins [13, 44].

**Fig. 1:**
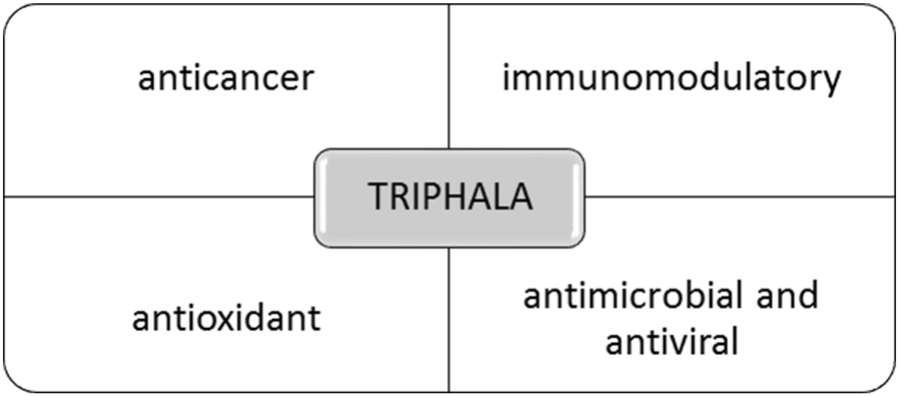
The properties of Triphala extracts

The major phytoconstituent of *T. bellerica*, *T. chebula* and *P. emblica* fruits is gallic acid, which is known to have a wide range of therapeutic activity, e.g. anti-atherosclerotic, hepatoprotective, cardioprotective, cytoprotective, cardiotonic, antimutagenic and antifungal [13, 45–47] (Fig. 1).

β-sitosterol, which is the main constituent of *T. chebula*, is an organic compound belonging to the family of phytosterols, whose chemical structure resembles cholesterol. It naturally occurs in various foods of plant origin and due to its specific properties it is widely used as a dietary supplement or food enrichment. β-sitosterol and phytosterols may affect lipid metabolism by inhibiting the absorption of cholesterol from the digestive tract. β-sitosterol affects sex hormone levels, which are used in the prevention of male pattern baldness, as well as in preventive treatment and post-exercise recovery. Some data suggest that β-sitosterol may increase the anabolic activity of testosterone; however, there are no clinical trials to confirm its action in humans. β-sitosterol has also been studied for its potential in reducing benign prostatic hyperplasia (BPH) [48, 49] and lowering high levels of blood-cholesterol [50].

## Therapeutic effects of TLP and its components

### Antimicrobial activity

Triphala is known to exert antibacterial action against a variety of Gram-positive and Gram-negative bacteria—e.g. *Streptococcus mutans*, a gram positive, anaerobic bacterium mostly found between adjacent teeth or in the deep crevices on occlusal of teeth, or *Helicobacter* pylori, the principal cause of inflammation in the gastric mucosa [51–53]. Worth mentioning, due to impaired immune system and thus inability to eliminate bacteria from the gastric mucosa, the bacteria-generated symptoms reach beyond the stomach and very often include abdominal pain, heartburn, belching or/and flatulence; the bacteria also affect lower GI motility and consequently result in constipation [52, 54].

Triphala is also an antiviral operator that acts against swine flu and other viruses, e.g. HSV-1, HIV-1 or cytomegalovirus [40, 55] or hepatitis B virus (HBV)-surface antigen [56].

### Antioxidant activity

Since antioxidants play an important role in protecting the human body against damages caused by reactive oxygen species (ROS), many studies have been devoted to uncovering the anti-oxidant potential of TLP. TLP possesses superoxide radical scavenging and hydroxyl radical-scavenging activity as shown in in vitro and in vivo studies [15, 57, 58]. Worth mentioning, the aqueous and alcoholic extracts of the individual constituents enhance the activation of macrophages due to their ability to scavenge free radicals and neutralize ROS [12, 59]. The alcoholic concentrate of *T. chebula* and *T. bellerica* extracts induce the production of ROS in macrophages mainly through the pro-phagocytic activity of gallic acid [25, 57, 60]. Similar results were reported in studies with *P. emblica* (amla) fruit extract [61]. Of note, amla is rich in antioxidants: flavonoids, carotenoids and vitamin C (in fact, it is considered as one of the richest sources of vitamin C) as proved in both in vitro and in vivo studies [9, 62]. The antioxidant effect of amla was assessed in rodents by measuring the activity of oxidative free radical scavenging enzymes, including the superoxide dismutase (SOD), catalase (CAT), glutathione peroxidase (GPX), as well as lipid peroxidation. In line, animals were allocated to one of the intervention groups to receive either an active compound of amla or deprenyl, a mono-amine oxidase B inhibitor used as a positive control. Amla and deprenyl increased the activity of SOD, CAT and GPX, and suppressed lipid peroxidation after 7 days of treatment, what confirmed the antioxidant efficacy of amla attributed to the presence of *P. emblica*, an active compound of tannoids [33]. In addition, phytochemicals found in *P. emblica* are also considered as good metal ion chelators as they hamper the oxidative cascades [63, 64].

### Hypolipidemic effect

A study by Manjunatha et al. [65] showed that volunteers given amla (500 mg/day) for 16 weeks had significantly improved HDL/LDL ratio and glucose tolerance, and reduced cholesterol level, in comparison to the control group receiving 500 mg/day of vitamin C. The antiatherosclerotic effects of *P. emblica* was also evaluated in rabbits fed with cholesterol-rich diet. After 60 days of supplementation with amla, the serum cholesterol, triglyceride, phospholipid and LDL levels were reduced by 82, 66, 77 and 90%, respectively. Importantly, the aortic plaques in treated animals were regressed suggesting a potent hypolipidemic action of *P. emblica* [35]. Another study [66] revealed that flavonoids extracted from amla exhibited intense hypolipidemic and hypoglycemic activities, and increased the level of hemoglobin in rats. Apparently, TLP is known to be one of the best ayurvedic medicines, with antioxidant and anti-aging properties.

### Anti-cancer activity

The major phytoconstituent of TLP—gallic acid, is believed to have a great impact on the anticancer properties of the polyherbal formulation of Ayurveda, as it inhibits cancer cell proliferation [45, 58]. Hence, it may be considered as a major determinant of antimutagenic and cytotoxic activity of TLP [45, 67]. In human colon cancer cells (HCT116) and stem cells (HCCSCs) the methanolic extracts of TLP exerted a dose-dependent antiproliferative properties and proapoptotic action independently of p53 status of the cells [67]. Moreover, treatment with TLP extract induced apoptosis via elevation of Bax/Bcl-2 ratio and suppressed c-Myc/cyclin D1 expression and therefore decreased cell proliferation and colony formation [59]. Recently, Gue et al. [68] showed that one of the constituents of TLP—*P. emblica*, delays mitotic progress, induces and accelerates apoptosis, and increases genomic instability in human adenocarcinoma cell line COLO320 in a dose-dependent manner and in human colon epithelial NCN460 cells. These results imply that *P. emblica* can protect human colon epithelial cells from genetic and mitotic damages and simultaneously indicate the necessity to evaluate its extract in depth in animal studies.

The antitumorigenic effect of TLP and its components were also determined in other cancer cell lines and in vivo studies. *P. emblica* and a preparation containing 50% of its natural extract given to mice significantly reduced ascites and Dalton’s lymphoma ascites cells-induced solid tumors, by inhibiting the cell cycle regulating enzymes such as CDC 25 phosphatase [69]. TRP also hampered benzo(a)pyrene-induced forestomach tumorigenesis, suppressed the growth of MCF-7 breast cancer cells, protected against radiation-induced oxidative damages and inhibited the proliferation of Capan-2 and BxPC-3 human pancreatic cancer cells through the activation of ERK, p53 and caspase-3 cascade [70–72].

The extract of TLP or its components hold potential as adjuvant therapy to the currently used anticancer agents, however currently available data warrant more extensive preclinical and clinical investigations.

### Immunomodulatory activity

It has been observed that through the range of biologically active compounds, e.g. gallic acid, ellagic acid, chebulinic acid etc., TLP can help reduce inflammation by lowering the expression of pro-inflammatory mediators [12, 73].

The alcohol extract of the dry ripe fruit of *T. chebula* protected cells from oxidative stress by inhibiting lipid peroxidation, increasing the levels of glutathione, superoxide dismutase and CAT, and by changing the mRNA expression of selected pro-inflammatory cytokines—IL-2, Il-10 and TNFα in vivo [74, 75]. TLP also improved the natural killer cell viability and antibody-dependent cellular cytotoxicity in Dalton’s lymphoma bearing mice [76]. The immunomodulatory characteristics of TLP have also been tested using carbon clearance test and delayed type hypersensitivity response in vivo (Foot Pad Swelling). TLP mega extract when administered orally (at low and high doses—100 and 500 mg/kg, respectively) increased the carbon clearance index, which was reflected in improved phagocytic function of mononuclear macrophage and nonspecific immunity. Moreover, the extract stimulated the production and release of T cells [77].

Many studies showed that modulation of NF-κB activity presents a promising target for the treatment of inflammation-related disorders [78] and since TLP can act via this pathway, it makes it a suitable option for the prevention or management of many diseases. In line, the study by Choi et al. [79] showed that gallic acid seen in TLP reduced the formation and release of pro-inflammatory mediators (TNFα and IL-6) by decreasing DNA binding to NF-κB in cells treated with lipopolysaccharides (LPS). In addition, the components of TLP and their metabolites, e.g. 4-*O*-methygallic acids and bellericanin, were able to suppress the expression and production of TNFα, IL-1β, IL-6, COX-2 and NO in RAW264.7 cells and primary macrophages stimulated with LPS, through the inhibition of redox-sensitive IκB kinase activity [24] and blocking NF-κB nuclear translocation [80–82]. The mixture of chebulagic, gallic and ellagic acids exerted immunomodulatory activity and thus makes *T. chebula* a potent plant material used to prevent cancer development [83]. TLP and its extracts have been useful in mimicking the action of neutrophils by preventing elevation of several cytokines, e.g. IL-4, IL-2 and IFN-γ. During the inflammatory stress the immunosuppressive activity of TLP is mainly linked to its inhibitory action on mitogen-induced T-lymphocyte proliferation and humoral and cell mediated immunity [12, 59].

Taken together, these observations indicate that the immunosuppressive effect of TLP and its extracts is attributed to decreased production of inflammatory mediators via the NF-κB pathway in experimental-induced inflammation and therefore may provide an alternative approach for the treatment of inflammatory and autoimmune diseases.

## Is TLP an effective remedy for improving GI symptoms?

Literature data does not provide any clear evidence showing the effect of TLP on the human digestive tract. Available studies indicate bidirectional activity of TLP (very often focusing on a particular component of TLP) as it is likely to either accelerate or slow down intestinal peristalsis. Mehmood et al. evaluated [16] the effect of *P. emblica* in constipation and indigestion in vivo and showed that *P. emblica* possessed prokinetic and laxative activities, and exerted spasmodic effect in the isolated ileum and jejunum of guinea pig and rabbit, respectively. The stimulatory action on the gut was supposedly mediated through the activation of muscarinic receptors. Another study was designed to evaluate the enteroprotective effect of TLP formulations in methotrexate-induced damages in rats. Different formulations of TLP solutions protected from intestinal injuries by improving epithelial cell integrity of tight junctions and restoring the brush border membrane of intestine, and decreasing the myeloperoxidase and xanthine oxidase level in the gut mucosa. The observed effect has been attributed to flavonoids, ellagic and gallic acids in TLP formulation. Depending on the proportions of *T. chebula*, *T. bellerica* and *P. emblica* the level of the injuries varied. It was suggested that unequal formulation of the ingredients in TLP i.e. the highest proportion of *P. emblica*, rather than its equal formulation provided better protection of intestinal microvilli [84].

Possible clinical application of TLP has also been assessed in patients with functional constipation. An open label, prospective, interventional and exploratory clinical trial conducted by Munshi et al. [85] demonstrated that 1 week treatment with TLP increases the average weekly bowel frequency by 64.4 and by 79.5% after 2 weeks. The increase in the mean bowel movement maintained 7 days after observatory period and was approximately 18% higher than that of baseline value. Another study showed that one of the constituents of TLP, *T. chebula*, ameliorated digestion and severe and continuous constipation as well as reduced intestinal cramps [9]. It has also been shown that the long-term (45 days) treatment with TLP improved the number, frequency and consistency of stools excreted per day and decreased the abdominal pain and bloating in healthy patients vs. control individuals who did not receive TLP [86]. No side effects in the treated group were reported [16, 85, 86].

It seems that TLP can be endorsed in the primary line treatment for patients with delayed GI motility, however several other studies [86, 87] indicated TLP as an anti-diarrheal agent thus disproving an earlier stated assumption. The anti-diarrheal effect of water and alcoholic solutions of TLP were examined in a rat model of castor oil-induced diarrhea. Each solution, independently of the dosage used in the study (200, 400 or 800 mg/kg) prolonged the whole GI transit and increased the weight of feces. In oral toxicity testing, aqueous and alcoholic solutions of TLP were viewed as effective and safe in the dosage up to 1750 mg/kg. It has been reported that the presence of phytochemicals in TLP, such as alkaloids, flavonoids, tannins, terpenes and sesquiterpenes could be responsible for the anti-diarrheal, antisecretory and spasmolytic effects of TLP [87].

The gut microbiota can protect the host from invading pathogens, maintain immune function and help absorb dietary nutrients. Quantitative and/or qualitative changes in the composition of intestinal microbiota can modulate the intestinal barrier function, particularly via the release of short chain fatty acids, and thus induce systemic immune response. There is a correlation between the abundance of specific microbial populations and the occurrence of GI symptoms e.g. an increase in *Bifidobacterium* is thought to be negatively correlated with intestinal symptoms such as bloating. Certain herbal extracts have proven its effectiveness in clinical trials in patients with FGID, therefore some attempts have already been made to evaluate the bioactivity of TLP and its extracts. Due to the presence of *P. emblica*, TLP inhibits the growth of pathogenic microorganisms in the intestines which may aggravate intestinal symptoms. Polyphenols in TLP can modulate the human gut microbiota by promoting the growth of beneficial *Bifidobacterium* and *Lactobacillus* species and inhibiting the growth of undesirable intestinal residents, such as *Escherichia coli*, which may induce the inflammatory reaction. Moreover, TLP-derived polyphenols e.g. chebulinic or ellagic acids, can be transformed by the human intestinal microbiota into various active metabolites, including urolithins, which modulate the inflammatory process by generating anti-inflammatory compounds and preventing from oxidative injuries of enterocytes [88]. The possible manipulation of gut microbiota by TLP extracts may represent a new strategy in the development of functional foods or nutraceuticals and become an effective way of restoring gut microbiome in patients with impaired lower GI function e.g. patients with IBS. Currently, there are no clinical trials assessing the effects of herbal formulations of TLP on clinical course of IBS thus it is difficult to determine the full value of TLP supplementation. Additional studies on human subjects are warranted given the recently-revealed enteroprotective, prokinetic and anti-bacterial properties of TLP in the GI tract.

## Concluding remarks

**Fig. 2:**
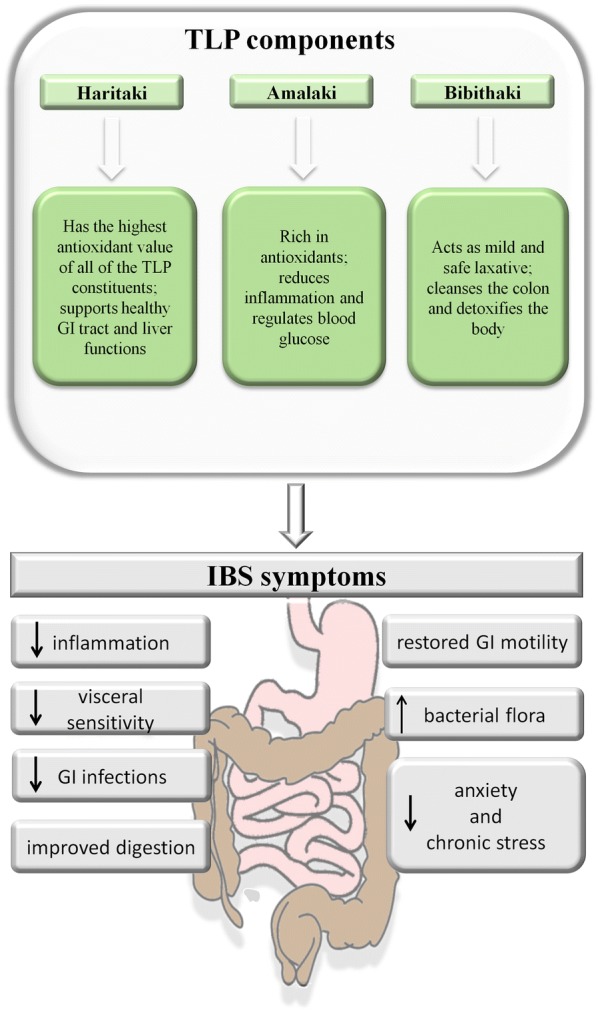
The TLP components and their effects on selected IBS symptoms

Medicinal plants still play a significant role in primary health care in many developing countries and have attracted renewed interest in developed countries over the last decades. Since natural products constitute a key source of drugs in medicine, the interest around them is increasing together with the number of studies trying to reveal their biological effects on humans. Since TLP is a multi-ingredient formulation it is likely that it could prove its effectiveness by modulating multiple targets, rather than acting on a single one. Various compelling preclinical in vitro and in vivo studies substantiated antioxidant, antimicrobial, immunomodulatory properties of TLP and its components, and proved its usefulness in an array of diseases, including hemorrhoid, skin disease, asthma, dysentery, uterine debility, anemia, diabetes, heart disease and others [15, 71, 77]. Although some attempts were made in order to determine the effects of TLP on lower GI tract, its action is still not well defined. Nevertheless, it seems that the herbal combination of TLP may bring benefits as adjuvant to currently applied treatment among patients with FGIDs, including IBS (Fig. 2).

It has been stated that allopathic medications represent a potential risk for human health and conversely their application may result in unintended side effects. None of these issues occur with the recommended dose of plant-based drugs and medicines whose mechanisms of action are well known and understood. Thus, it is essential to deflect attention away from ordinary allopathic medications to customary plant based medications. Further research focusing on sub-atomic level of these plants is needed. This analysis could be a stage towards assessing the pharmacological properties of TLP and its three distinct constituents.

In vitro and in vivo animal studies undeniably indicate that TLP may have anti-inflammatory, antibiotic, and anti-cancer properties in the GI tract. However, the only clinical trial determining the effect of TLP on the GI tract has been carried out to prove its application in dentistry [89].

Although the limited data of clinical research of the role of TLP in the GI tract does not allow drawing a firm conclusion about its effectiveness for IBS patients, nevertheless there are some premises, which indicate its potential. By virtue of its laxative qualities, TLP would not be helpful for diarrhea-predominant IBS (IBS-D) patients but its application is worth considering to patients with IBS-C. It is also possible that TLP can alleviate other symptoms experienced by patients, through the interaction of each constituent of TLP and gut microbiota; therefore supplementation of TLP herbal formulations alone or along with other probiotics should be taken into consideration in an ongoing clinical studies.

Contrary to pharmaceutical laxatives, which tend to stimulate the bowel, TLP has a regulating effect and can be used long-term. The large intestine is permanently exposed to various toxins, parasites, etc. therefore, it is important to provide adequate bowel cleansing. Literature data indicate that TLP acts as a colon cleanser, which helps to clean the waste matter from the lower GI tract and improve its proper functioning [12, 88]. To sum up, TLP can be particularly helpful if constipation is a symptom, but it can also be useful in some cases of alternating constipation.

Relevant cell culture and animal studies, and rationally designed clinical trials are required to uncover the potential of TLP, understand its mechanism of action and determine permissible dose that could be incorporated along with patient’s therapy and assess side effects, which may appear over its longer administration.

## Abbreviations

CAT: catalase
Cdc25: dual-specificity phosphatase
Cdk: cyclin dependent kinases
FBDSI: functional bowel disorder severity index
FGIDs: functional gastrointestinal disorders
GI: gastrointestinal
GPX: glutathione peroxidase
HBV: hepatitis B virus
HSV-1: herpes simplex virus 1
HPTLC: high performance thin layer chromatography
IBS: irritable bowel syndrome
LPS: lipopolysaccharide
MCs: mast cells
SOD: superoxide dismutase
TLP: Triphala

## References

[1] Spiller R, Aziz Q, Creed F, Emmanuel A, Houghton L, Hungin P (2007). Guidelines on the irritable bowel syndrome: mechanisms and practical management. Gut.

[2] Grundmann O, Yoon SL (2014). Complementary and alternative medicines in irritable bowel syndrome: an integrative view. World J Gastroenterol.

[3] Barbara G, Zecchi L, Barbaro R, Cremon C, Bellacosa L, Marcellini M (2012). Mucosal permeability and immune activation as potential therapeutic targets of probiotics in irritable bowel syndrome. J Clin Gastroenterol.

[4] Kilpatrick LA, Gupta A, Love AD, Labus JS, Alaverdyan M, Tillisch K (2015). Neurobiology of psychological resilience in irritable bowel syndrome (IBS) and inflammatory bowel disease (IBD) patients. Gastroenterology.

[5] Lauche R, Kumar S, Hallmann J, Lüdtke R, Rampp T, Dobos G (2016). Efficacy and safety of Ayurvedic herbs in diarrhoea-predominant irritable bowel syndrome: a randomised controlled crossover trial. Complement Ther Med.

[6] Joos S, Rosemann T, Szecsenyi J, Hahn EG, Willich SN, Brinkhaus B (2006). Use of complementary and alternative medicine in Germany—a survey of patients with inflammatory bowel disease. BMC Complement Altern Med.

[7] Wasilewski A, Zielińska M, Storr M, Fichna J (2015). Beneficial effects of probiotics, prebiotics, synbiotics, and psychobiotics in inflammatory bowel disease. Inflamm Bowel Dis.

[8] Basturk A, Artan R, Yilmaz A (2016). Efficacy of synbiotic, probiotic, and prebiotic treatments for irritable bowel syndrome in children: a randomized controlled trial. Turkish J Gastroenterol.

[9] Rani B, Prasad M, Kumar R, Vikram Y, Kachhawa GR, Sharma S (2013). Triphala: a versatile counteractive assortment of ailments. Int J Pharm Chem Sci.

[10] Kumar NS, Nair AS, Nair AM, Murali M (2016). Pharmacological and therapeutic effects of triphala—a literature review. J Pharmacogn Phytochem JPP.

[11] Gupta M (2010). Therapeutic uses of the polyherbal drug Triphala in geriatric diseases. Int J Pharma Bio Sci.

[12] Belapurkar P, Goyal P, Tiwari-Barua P (2014). Immunomodulatory effects of triphala and its individual constituents: a review. Indian J Pharm Sci.

[13] Pharmacy F, St P, Ake M, Thani P (2016). HPLC-MS profiles and quantitative analysis of triphala formulation. BHST.

[14] Ghosh A, Das BK, Roy A, Mandal B, Chandra G (2008). Antibacterial activity of some medicinal plant extracts. J Nat Med.

[15] Chandran U, Mehendale N, Tillu G, Patwardhan B (2015). Network pharmacology of ayurveda formulation Triphala with special reference to anti-cancer property. Comb Chem High Throughput Screen.

[16] Mehmood MH, Rehman A, Rehman N, Gilani A-H (2013). Studies on prokinetic, laxative and spasmodic activities of *Phyllanthus emblica* in experimental animals. Phyther Res.

[17] Manosroi A, Jantrawut P, Akazawa H, Akihisa T, Manosroi J (2010). Biological activities of phenolic compounds isolated from galls of *Terminalia chebula* Retz. (Combretaceae). Nat Prod Res.

[18] Lee H-S, Koo Y-C, Suh HJ, Kim K-Y, Lee K-W (2010). Preventive effects of chebulic acid isolated from *Terminalia chebula* on advanced glycation endproduct-induced endothelial cell dysfunction. J Ethnopharmacol.

[19] Lu K, Chakroborty D, Sarkar C, Lu T, Xie Z, Liu Z (2012). Triphala and its active constituent chebulinic acid are natural inhibitors of vascular endothelial growth factor-A mediated angiogenesis. PLoS ONE.

[20] Shen Y-C, Juan C-W, Lin C-S, Chen C-C, Chang C-L (2017). Neuroprotective effect of *Terminalia chebula* extracts and ellagic acid in pc12 cells. Afr J Tradit Complement Altern Med.

[21] Zamora-Ros R, Knaze V, Rothwell JA, Hémon B, Moskal A, Overvad K (2016). Dietary polyphenol intake in Europe: the European prospective investigation into cancer and nutrition (EPIC) study. Eur J Nutr.

[22] Chokotho L, van Hasselt E (2005). The use of tannins in the local treatment of burn wounds—a pilot study. Malawi Med J.

[23] Na H-J, Lee G, Oh H-Y, Jeon K-S, Kwon H-J, Ha K-S (2006). 4-*O*-Methylgallic acid suppresses inflammation-associated gene expression by inhibition of redox-based NF-kappaB activation. Int Immunopharmacol.

[24] Jadon A, Bhadauria M, Shukla S (2007). Protective effect of *Terminalia belerica* Roxb. and gallic acid against carbon tetrachloride induced damage in albino rats. J Ethnopharmacol.

[25] Tam PE, Hinsdill RD (1990). Screening for immunomodulators: effects of xenobiotics on macrophage chemiluminescence in vitro. Fundam Appl Toxicol.

[26] Shay J, Elbaz HA, Lee I, Zielske SP, Malek MH, Hüttemann M (2015). Molecular mechanisms and therapeutic effects of (−)-epicatechin and other polyphenols in cancer, inflammation, diabetes, and neurodegeneration. Oxid Med Cell Longev..

[27] Singh A, Demont A, Actis-Goretta L, Holvoet S, Lévêques A, Lepage M (2014). Identification of epicatechin as one of the key bioactive constituents of polyphenol-enriched extracts that demonstrate an anti-allergic effect in a murine model of food allergy. Br J Nutr.

[28] Cox CJ, Choudhry F, Peacey E, Perkinton MS, Richardson JC, Howlett DR (2015). Dietary (−)-epicatechin as a potent inhibitor of βγ-secretase amyloid precursor protein processing. Neurobiol Aging.

[29] Actis-Goretta L, Lévèques A, Rein M, Teml A, Schäfer C, Hofmann U (2013). Intestinal absorption, metabolism, and excretion of (−)-epicatechin in healthy humans assessed by using an intestinal perfusion technique. Am J Clin Nutr.

[30] Singh DP, Govindarajan R, Rawat AKS (2008). High-performance liquid chromatography as a tool for the chemical standardisation of Triphala—an ayurvedic formulation. Phytochem Anal.

[31] Guo X, Wang X (2016). *Phyllanthus emblica* fruit extract activates spindle assembly checkpoint, prevents mitotic aberrations and genomic instability in human colon epithelial NCM460 cells. Int J Mol Sci.

[32] Hayat Khan K, Khan K (2009). Roles of *Emblica officinalis* in medicine—a review. Bot Res Int.

[33] Bhattacharya A, Chatterjee A, Ghosal S, Bhattacharya SK (1999). Antioxidant activity of active tannoid principles of *Emblica officinalis* (amla). Indian J Exp Biol.

[34] Bahrami HR, Hamedi S, Salari R, Noras M (2016). Herbal medicines for the management of irritable bowel syndrome: a systematic review. Electron Physician.

[35] Mathur R, Sharma A, Dixit VP, Varma M (1996). Hypolipidaemic effect of fruit juice of *Emblica officinalis* in cholesterol-fed rabbits. J Ethnopharmacol.

[36] Majeed M, Bhat B, Jadhav AN, Srivastava JS, Nagabhushanam K (2009). Ascorbic acid and tannins from *emblica officinalis* Gaertn. FruitssA Revisit. J Agric Food Chem.

[37] Wazir A, Mehjabeen, Jahan N, Sherwani SK, Ahmad M (2014). Antibacterial, antifungal and antioxidant activities of some medicinal plants. Pak J Pharm Sci.

[38] Tarwadi K, Agte V (2007). Antioxidant and micronutrient potential of common fruits available in the Indian subcontinent. Int J Food Sci Nutr.

[39] Barthakur NN, Arnold NP (1991). Nutritive value of the chebulic myrobalan (*Terminalia chebula* Retz.) and its potential as a food source. Food Chem.

[40] Ahn M-J, Kim CY, Lee JS, Kim TG, Kim SH, Lee C-K (2002). Inhibition of HIV-1 integrase by galloyl glucoses from *Terminalia chebula* and flavonol glycoside gallates from *Euphorbia Pekinensis*. Planta Med.

[41] Juang L-J, Sheu S-J (2005). Chemical identification of the sources of commercial Fructus Chebulae. Phytochem Anal.

[42] Cock IE (2015). The medicinal properties and phytochemistry of plants of the genus Terminalia (Combretaceae). Inflammopharmacology.

[43] Walden R, Tomlinson B, Benzie IFF, Wachtel-Galor S (2011). Cardiovascular disease. Herbal medicine: biomolecular and clinical aspects, Chapter 16.

[44] Pawar V, Lahorkar P, Anantha Narayana DB (2009). Development of a RP-HPLC method for analysis of Triphala curna and its applicability to test variations in Triphala curna preparations. Indian J Pharm Sci.

[45] Kaur S, Michael H, Arora S, Härkönen PL, Kumar S (2005). The in vitro cytotoxic and apoptotic activity of Triphala—an Indian herbal drug. J Ethnopharmacol.

[46] Ponnusankar S, Pandit S, Babu R, Bandyopadhyay A, Mukherjee PK (2011). Cytochrome P450 inhibitory potential of Triphala—a rasayana from ayurveda. J Ethnopharmacol.

[47] Mukherjee PK, Harwansh RK, Bahadur S, Banerjee S, Kar A, Chanda J (2016). Development of ayurveda—tradition to trend. J Ethnopharmacol.

[48] Wilt TJ, Ishani A, MacDonald R, Stark G, Mulrow CD, Lau J (1999). Beta-sitosterols for benign prostatic hyperplasia. Cochrane Database Syst Rev..

[49] Shin T, Jeong H, Kim D, Kim S, Lee J, Kim D (2001). Inhibitory action of water soluble fraction of *Terminalia chebula* on systemic and local anaphylaxis. J Ethnopharmacol.

[50] Rudkowska I, AbuMweis SS, Nicolle C, Jones PJH (2008). Cholesterol-lowering efficacy of plant sterols in low-fat yogurt consumed as a snack or with a meal. J Am Coll Nutr.

[51] Malekzadeh F, Ehsanifar H, Shahamat M, Levin M, Colwell R (2001). Antibacterial activity of black myrobalan (*Terminalia chebula* Retz) against Helicobacter pylori. Int J Antimicrob Agents.

[52] Malfertheiner P, Me F, Graud Â, O’morainà C, Hungin APS, Jones R (2007). Current concepts in the management of *Helicobacter pylori* infection: the Maastricht III consensus report. Gut.

[53] Jagtap AG, Karkera SG (1999). Potential of the aqueous extract of *Terminalia chebula* as an anticaries agent. J Ethnopharmacol.

[54] Xiong F, Xiong M, Ma Z, Huang S, Li A, Liu S (2016). Imported from lack of association found between *Helicobacter pylori* infection and diarrhea predominant irritable bowel syndrome: a multicenter retrospective study. Gastroenterol Res Pract.

[55] Kim TG, Kang SY, Jung KK, Kang JH, Lee E, Han HM (2001). Antiviral activities of extracts isolated from *Terminalis chebula* Retz., *Sanguisorba officinalis* L., *Rubus coreanus* Miq. and *Rheum palmatum* L. against hepatitis B virus. Phytother Res.

[56] Kannan M, Rajendran P, Pratap C, Vedha V, Ashok G, Anushka S (2011). Inhibition of hepatitis B virus DNA polymerase and modulation of TH1 & TH2 cytokine secretion by three Indian medicinal plants and its correlation with antiviral properties. J Pharm Res.

[57] Sabu MC, Kuttan R (2002). Anti-diabetic activity of medicinal plants and its relationship with their antioxidant property. J Ethnopharmacol.

[58] Sandhya T, Mishra KP (2006). Cytotoxic response of breast cancer cell lines, MCF 7 and T 47 D to triphala and its modification by antioxidants. Cancer Lett.

[59] Kovačević N, Čolić M, Backović A, Došlov-Kokoruš Z (2006). Immunomodulatory effects of the methanolic extract of *Epimedium alpinum* in vitro. Fitoterapia.

[60] Houde V, Grenier D, Chandad F (2006). protective effects of grape seed proanthocyanidins against oxidative stress induced by lipopolysaccharides of periodontopathogens. J Periodontol.

[61] Suja RS, Nair AMC, Sujith S, Preethy J, Deepa AK (2009). Evaluation of immunomodulatory potential’ of *Emblica officinalis* fruit pulp extract in mice. Indian J Anim Res.

[62] Ahmad I, Mehmood Z, Mohammad F (1998). Screening of some Indian medicinal plants for their antimicrobial properties. J Ethnopharmacol.

[63] Kumaran A, Karunakaran RJ (2006). Nitric oxide radical scavenging active components from *Phyllanthus emblica* L. Plant Foods Hum Nutr.

[64] Niwano Y, Saito K, Yoshizaki F, Kohno M, Ozawa T (2011). Extensive screening for herbal extracts with potent antioxidant properties. J Clin Biochem Nutr.

[65] Manjunatha S, Jaryal AK, Bijlani RL, Sachdeva U, Gupta SK (2001). Effect of Chyawanprash and vitamin C on glucose tolerance and lipoprotein profile. Indian J Physiol Pharmacol.

[66] Anila L, Vijayalakshmi NR (2000). Beneficial effects of flavonoids from *Sesamum indicum*, *Emblica officinalis* and *Momordica charantia*. Phytother Res.

[67] Vadde R, Radhakrishnan S, Reddivari L, Vanamala JKP. Triphala Extract suppresses proliferation and induces apoptosis in human colon cancer stem cells via suppressing c-Myc/Cyclin D1 and elevation of Bax/Bcl-2 ratio. Biomed Res Int. 2015. Article id: 649263. 10.1155/2015/649263 PMC4488090 26167492

[68] Guo X, Ni J, Liu X, Xue J, Wang X (2013). *Phyllanthus emblica* L. fruit extract induces chromosomal instability and suppresses necrosis in human colon cancer cells. Int J Vitam Nutr Res.

[69] Jose JK, Kuttan G, Kuttan R (2001). Antitumour activity of *Emblica officinalis*. J Ethnopharmacol.

[70] Deep G, Dhiman M, Rao AR, Kale RK (2005). Chemopreventive potential of Triphala (a composite Indian drug) on benzo(a)pyrene induced forestomach tumorigenesis in murine tumor model system. J Exp Clin Cancer Res.

[71] Sandhya T, Lathika KM, Pandey BN, Mishra KP (2006). Potential of traditional ayurvedic formulation, Triphala, as a novel anticancer drug. Cancer Lett.

[72] Shi Y, Sahu RP, Srivastava SK (2008). Triphala inhibits both in vitro and in vivo xenograft growth of pancreatic tumor cells by inducing apoptosis. BMC Cancer.

[73] Srikumar R, Parthasarathy NJ, Manikandan S, Muthuvel A, Rajamani R, Sheeladevi R (2007). Immunomodulatory effect of Triphala during experimentally induced noise stress in albino rats. J Heal Sci.

[74] Aher V, Wahi A (2011). Immunomodulatory activity of alcohol extract of *Terminalia chebula* retz combretaceae. Trop J Pharm Res.

[75] Liu Y, Bao L, Xuan L, Song B, Lin L, Han H (2015). Chebulagic acid inhibits the LPS-induced expression of TNF-α and IL-1β in endothelial cells by suppressing MAPK activation. Exp Ther Med.

[76] Baliga MS (2010). Triphala, ayurvedic formulation for treating and preventing cancer: a review. J Altern Complement Med.

[77] Gowda DV, Muguli G, Rangesh PR, Deshpande RD (2012). Phytochemical and pharmacological actions of triphala: ayurvedic formulation—a review. Int J Pharm Sci Rev Res.

[78] Monkkonen T, Debnath J (2017). Inflammatory signaling cascades and autophagy in cancer. Autophagy.

[79] Choi K-C, Lee Y-H, Jung MG, Kwon SH, Kim M-J, Jun WJ (2009). Gallic acid suppresses lipopolysaccharide-induced nuclear factor-κB signaling by preventing RelA acetylation in A549 lung cancer cells. Mol Cancer Res.

[80] Zhao L, Zhang S-L, Tao J-Y, Pang R, Jin F, Guo Y-J (2008). Preliminary exploration on anti-inflammatory mechanism of corilagin (beta-1-*O*-galloyl-3,6-(R)-hexahydroxydiphenoyl-d-glucose) in vitro. Int Immunopharmacol.

[81] Karlsson S, Nånberg E, Fjaeraa C, Wijkander J (2010). Ellagic acid inhibits lipopolysaccharide-induced expression of enzymes involved in the synthesis of prostaglandin E2 in human monocytes. Br J Nutr.

[82] Ho H-H, Chang C-S, Ho W-C, Liao S-Y, Wu C-H, Wang C-J (2010). Anti-metastasis effects of gallic acid on gastric cancer cells involves inhibition of NF-κB activity and downregulation of PI3K/AKT/small GTPase signals. Food Chem Toxicol.

[83] Sehar I, Kaul A, Bani S, Pal HC, Saxena AK (2008). Immune up regulatory response of a non-caloric natural sweetener, stevioside. Chem Biol Interact.

[84] Nariya M, Shukla V, Jain S, Ravishankar B (2009). Comparison of enteroprotective efficacy of triphala formulations (Indian herbal drug) on methotrexate-induced small intestinal damage in rats. Phyther Res.

[85] Munshi R, Bhalerao S, Rathi P, Kuber VV, Nipanikar SU, Kadbhane KP (2011). An open-label, prospective clinical study to evaluate the efficacy and safety of TLPL/AY/01/2008 in the management of functional constipation. J Ayurveda Integr Med.

[86] Mukherjee P, Rai S, Bhattacharyya S (2007). Clinical study of ‘Triphala’—a Weill known phytomedicine from India. Iran J Pharmacol Ther..

[87] Mehmood MH, Siddiqi HS, Gilani AH (2010). The antidiarrheal and spasmolytic activities of *Phyllanthus emblica* are mediated through dual blockade of muscarinic receptors and Ca2+ channels. J Ethnopharmacol.

[88] Selma MV, Beltrán D, Luna MC, Romo-Vaquero M, García-Villalba R, Mira A (2017). Isolation of human intestinal bacteria capable of producing the bioactive metabolite isourolithin a from ellagic acid. Front Microbiol.

[89] Bhavikatti SK, Dhamija R, Prabhuji MLV, Emblica A (2015). Review article Triphala: envisioning its role in dentistry. Int Res J Pharm.

